# Identifying New COVID-19 Receptor Neuropilin-1 in Severe Alzheimer’s Disease Patients Group Brain Using Genome-Wide Association Study Approach

**DOI:** 10.3389/fgene.2021.741175

**Published:** 2021-10-21

**Authors:** Key-Hwan Lim, Sumin Yang, Sung-Hyun Kim, Jae-Yeol Joo

**Affiliations:** Neurodegenerative Disease Research Group, Korea Brain Research Institute, Daegu, South Korea

**Keywords:** SARS-CoV-2, Neuropilin-1, Alzheimer’s disease, genome-wide association study (GWAS), gene expression

## Abstract

Recent preclinical studies show that Neuropilin-1 (NRP1), which is a transmembrane protein with roles in neuronal development, axonal outgrowth, and angiogenesis, also plays a role in the infectivity of severe acute respiratory syndrome coronavirus 2 (SARS-CoV-2). Thus, we hypothesize that NRP1 may be upregulated in Alzheimer’s disease (AD) patients and that a correlation between AD and SARS-CoV-2 NRP1-mediated infectivity may exist as angiotensin converting enzyme 2 (ACE2). We used an AD mouse model that mimics AD and performed high-throughput total RNA-seq with brain tissue and whole blood. For quantification of NRP1 in AD, brain tissues and blood were subjected to Western blotting and real-time quantitative PCR (RT-qPCR) analysis. *In silico* analysis for NRP1 expression in AD patients has been performed on human hippocampus data sets. Many cases of severe symptoms of COVID-19 are concentrated in an elderly group with complications such as diabetes, degenerative disease, and brain disorders. Total RNA-seq analysis showed that the *Nrp1* gene was commonly overexpressed in the AD model. Similar to ACE2, the NRP1 protein is also strongly expressed in AD brain tissues. Interestingly, *in silico* analysis revealed that the level of expression for NRP1 was distinct at age and AD progression. Given that NRP1 is highly expressed in AD, it is important to understand and predict that NRP1 may be a risk factor for SARS-CoV-2 infection in AD patients. This supports the development of potential therapeutic drugs to reduce SARS-CoV-2 transmission.

## Introduction

Severe acute respiratory syndrome coronavirus 2 (SARS-CoV-2) is being evaluated as a third-high-risk contagious infection (Hu et al., 2020). People are still highly vulnerable to the ongoing and life-threatening COVID-19 pandemic, as FDA-authorized vaccines or beneficial treatments remain unavailable (Singh et al., 2020). The risk of severe complications that are eventually associated with high mortality is indicated in older people (Carstensen et al., 2020). Moreover, a bidirectional interrelation between neurological complications and COVID-19 is extensively reported (Verkhratsky et al., 2020).

Age-dependent vulnerability to SARS-CoV-2 has been associated with concomitant symptomatic infections (Liu et al., 2020; Wu et al., 2020). Alzheimer’s disease (AD) is a highly destructive neurodegenerative disorder that mostly affects the elderly and is characterized by a progressive cognitive decline (Masters et al., 2015). Although various hypotheses have been proposed to explain its multifactorial properties (Liu et al., 2019), the exact mechanism and related features of AD remain obscure. An analysis of 627 patients suggests that AD is a risk factor for SARS-CoV-2 infection (Bianchetti et al., 2020).

Angiotensin converting enzyme 2 (ACE2) is required for SARS-CoV-2 infection. Recently, it is reported that the *Ace2* gene and protein expression are elevated in AD patients compared with in normal elderly individuals (Ding et al., 2020; Lim et al., 2020; Rahman et al., 2020). Consistent with these results, an increase in ACE2 expression results in an increased susceptibility to SARS-CoV-2 infection in elderly patients with AD. Furthermore, a recent study suggests that the transmembrane protein Neuropilin-1 (NRP1) also plays a role in SARS-CoV-2 infection (Daly et al., 2020; Mayi et al., 2021). Biochemical experiments and X-ray crystallography show that NRP1 strongly interacts with a polybasic sequence on the spike protein of SARS-CoV-2, which fits the C-end rule region (CendR) required for NRP1-peptide interaction (Daly et al., 2020; Song et al., 2020). NRP1 depletion with RNAi targeting Nrp1 mRNA inhibits the binding of the SARS-CoV-2 spike protein to NRP1 and, consequently, decreases the rate of viral infection (Daly et al., 2020; Song et al., 2020). In addition, a monoclonal antibody against the b1b2 domain of NRP1 reduces the infectivity of SARS-CoV-2 lentiviral pseudo-particles (Cantuti-Castelvetri et al., 2020). NRP1 is a neuronal receptor associated with the regulation of neurite outgrowth through the binding of vascular endothelial growth factor (VEGF) (Abdullah et al., 2020). When NRP1 is activated by CendR, which is a peptide R/KXXR/K motif contained within C-terminal domains, it enables cells to internalize ligands, such as viruses, containing the motif (Teesalu et al., 2009). Furthermore, NRP1 is expressed in the central nervous system, including the brain olfactory-related regions in which SARS-CoV-2 entry may occur, thereby facilitating COVID-19 infection (Davies et al., 2020).

Thus, we hypothesize that, in addition to ACE2, NRP1 expression might be upregulated in the brains of elderly AD patients. In this study, molecular characterization via high-throughput analysis and biochemical assays reveals that NRP1 is highly expressed in AD, which suggests that NRP1 may be a potential genetic therapy target in AD patients with COVID-19.

## Materials and Methods

### Animals

Five × FAD transgenic mice were purchased from the Jackson Laboratory. All animal experiments performed in this study were reviewed and approved by the IACUC committee at the Korea Brain Research Institute (IACUC-20-00018).

### Total RNA Sequencing and Human *in silico* Analysis

The data analysis of total RNA-seq from the mouse cortex was performed as previously described in Lim and Joo (2020). Briefly, the brain was extracted from 6-month-old wild-type (WT) and 5×FAD mice and cortex isolated to prepare the pure RNA and total RNA-seq library. RNA-seq libraries were prepared using the TruSeq Stranded Total RNA LT Sample Prep Kit (Illumina Sample Preparation Guide) from isolated mRNA. To profile the insert length of libraries, we used the Agilent 2100 Bioanalyzer, and constructed libraries were sequenced from HiSeq^TM^4000 platform (Illumina, United States). Then, converted nucleotide sequences using HiSeq^TM^4000 were sorted and the dirty reads filtered from the raw reads. RNA-seq data was accessible using Gene Expression Omnibus (GEO) accession number GSE147792. *In silico* data analysis was performed using the Affymetrix Human Genome U133 Plus 2.0 Array (Lim et al., 2020). The GSE1297 data sets were derived from human hippocampus and GSE4226 data sets were derived from human peripheral blood mononuclear cells (PBMCs) in normal and AD patients.

### RNA Isolation

Total RNA isolation was performed with the mouse cortex according to TRIzol using the commercial protocol. First, phenol-based TRIzol (Invitrogen) is added in the cortex tissue tube for homogenizing. Then, it is separated into three phases by chloroform for the collect only RNA dissolved aqueous phase except the DNA and protein precipitated phases. An equal volume of isopropanol was used to precipitate RNA. After centrifugation, supernatant was discarded, and it was washed with prechilled 75% ethanol once. RNA was dehydrated and crystalized without organic compound contamination and eluted with nuclease-free water. RNA was then denatured in the 65°C heat block for 10 min. The procedure was performed without RNase contamination.

### Complementary DNA Synthesis

Isolated total RNA was synthesized into complementary DNA (cDNA) following the manufacturer’s protocol of High-Capacity cDNA Reverse Transcription Kits (Applied Biosystems). Template RNA (2 μg) was prepared to synthesize a single reaction, and reverse transcription kit components were premixed. The premixture contains 10 × RT buffer, 25 × dNTP mix (4 mM), 10 × RT Random Primers, MultiScribe Reverse Transcriptase (50 U), RNase inhibitor, and nuclease-free water for adjusting the total volume for the reaction. Gently mixed template RNA and an equal volume of premixture was placed in the thermal cycler. The condition for reverse transcription was suggested as optimized temperature and time: 25°C for 10 min, 37°C for 120 min, and 85°C for 5 min.

### Real-Time Quantitative PCR

Real-time quantitative PCR (RT-qPCR) was performed according to commercial protocol using SYBR Green PCR Master Mix (Applied Biosystems). Primers employed were *Nrp1* forward, 5′ CCTCACATTGGGCGTTATTG 3′, reverse, 5′ CACTGTAGTTGGCTGAGAAAC 3′; *Gapdh* forward, 5′ AGGTCGGTGTGAACGGATTT 3′, reverse, 5′ TGTAGACCATGTAGTTGAGG 3′. Each reaction contains SYBR Green PCR Master Mix, Template cDNA, and forward and reverse primer and is adjusted with nuclease-free water.

### Western Blot

Protein was extracted from the mouse cortex and mixed with sample buffer (5% 2-mercaptoethanol) and boiled at 100°C for denaturation. Protein samples were loaded on 4–15% gradient gel (Bio-Rad) to separate by size through the vertical SDS-PAGE system. Antibodies used for immune-blot analysis were anti-Neuropilin-1 (abcam, 1:1000) and anti-β-actin (BETHYL, 1:10000). Images were acquired by ChemiDoc MP imaging system (Bio-Rad).

## Results

### High-Throughput Analysis of *Nrp1* Expression in Alzheimer’s Disease

**FIGURE 1:**
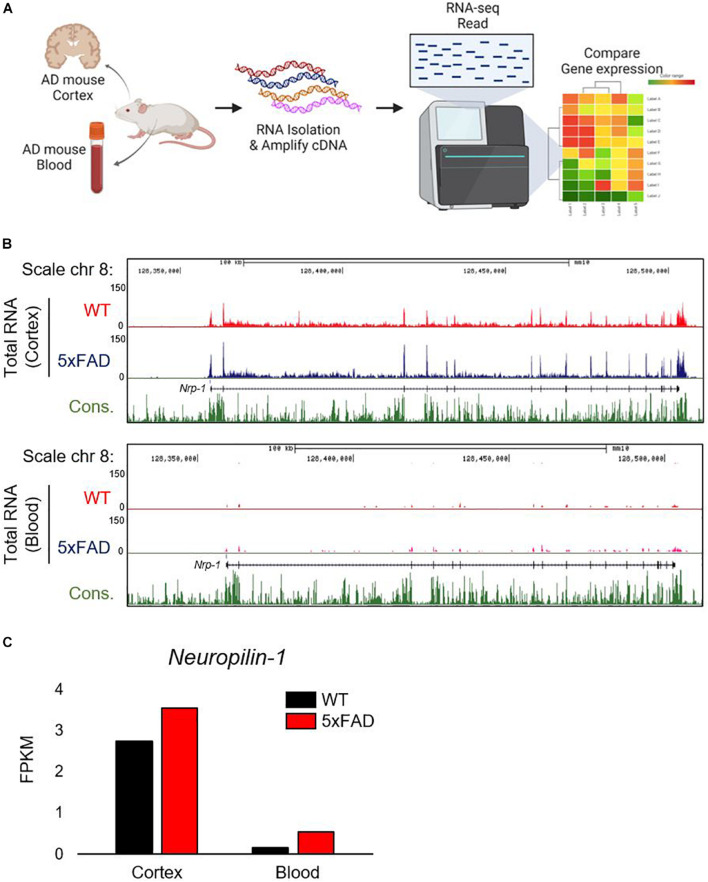
Correlation between *Nrp1* gene and protein expression in AD brain. **(A)** Graphic representation of *Nrp1* gene expression in the cortex of five familial AD mutations (5×FAD) mice, which are used as a murine model of AD. **(B)** The representation is shown on the UCSC genome browser following total RNA-seq. **(C)** *Nrp1* gene expression levels are increased by 129% in 5×FAD cortex compared with control WT cortex.

Given that the gene expression of ACE2 is upregulated in the brains of patients with AD and may be associated with the mortality rate from COVID-19 in the elderly (Fu et al., 2020; Lim et al., 2020), we hypothesize that NRP1, which codes for a newly recognized SARS-CoV-2 spike receptor, may be also increased in AD patients. To assess *Nrp1* gene expression in AD, we first used a murine model that mimics AD and performed total RNA-seq using mouse brain tissue and whole blood. Total RNA-seq was analyzed by the HiSeq^TM^4000 platform (Illumina, United States) (Figure 1A). We applied the *Nrp1* gene expression level in the brain and blood from AD and WT and then mapped the sequencing reads (Figure 1B). The track of *Nrp1* gene was displayed with University of California, Santa Cruz (USCS) genome browser (Figure 1B). Interestingly, total RNA-seq analysis revealed upregulation of *Nrp1* gene expression in the brain of the AD model compared to WT (Figure 1B), and *Nrp1* fragments per kb per million reads values are increased in the AD model brain as well (Figure 1C). Although *Nrp1* gene expression was increased by 319% in AD blood compared with WT blood, the endogenous expression levels of *Nrp1* in the blood were significantly lower than those in the brain (Figures 1B,C). Collectively, our total RNA-seq results show that *Nrp1* is preferentially expressed in the brain and upregulated in the brains of AD mice.

### *Nrp1* Is Upregulated in Alzheimer’s Disease Brain

*Nrp1* is abundantly expressed in the neurons and plays an important role for axon guidance, regeneration, neuronal plasticity, or various human diseases, such as epilepsy and seizure (Kumanogoh and Kikutani, 2013).

**FIGURE 2:**
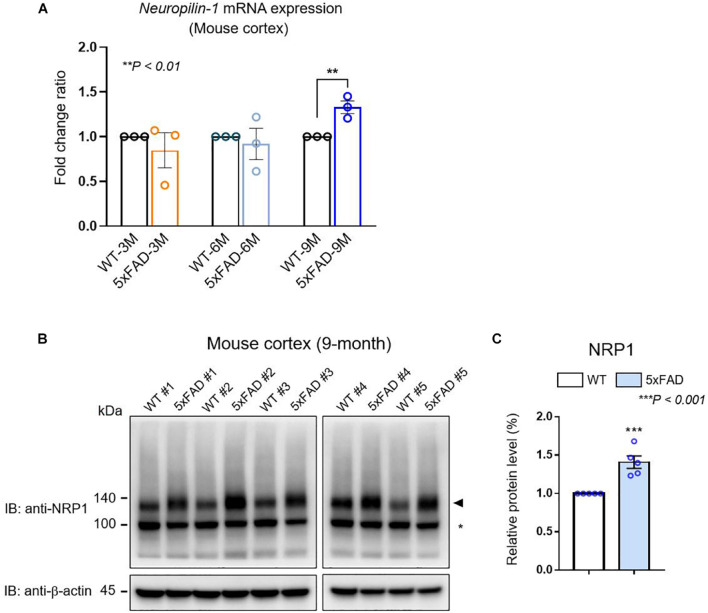
The expression of NRP1 in mouse AD brain. **(A)** RT-qPCR analysis showing the *Nrp1* mRNA expression levels in the cortex of WT and 5×FAD mice. *Nrp1* mRNA expression is significantly increased in 9-month-old 5×FAD cortex compared with that in WT cortex. No significant differences are observed in the early disease stages of 5×FAD mice (3 and 6 months). The data are shown as the mean ± standard error of the mean (SEM) from *n* = 3 mice per group; statistical differences were assessed using unpaired *t*-test. **(B)** Representative Western blot analyzing the NRP1 protein levels in 5×FAD brains. Endogenous NRP1 is highly expressed in 9-month-old 5×FAD brains compared with that in the WT brain. β-actin was used as a loading control. The arrowhead indicates the NRP1 protein, and the asterisk indicates a non-specific band (*n* = 5 mice per group). **(C)** NRP1 Western blot band intensity measured by ImageJ 1.50i software (*n* = 5 mice per group). Statistical differences were assessed using unpaired *t*-test.

We confirmed *Nrp1* gene expression in both WT and AD model mouse brains through the total RNA-seq (Figure 1). To further analyze *Nrp1* expression during AD progression, we measured the Nrp1 mRNA levels in 3- to 9-month-old AD brains. RT-qPCR revealed an approximately 145% increase in *Nrp1* mRNA expression in 9-month-old AD brains compared with that in WT brains (Figure 2A). In addition, NRP1 protein expression was also significantly increased in 9-month-old AD brains compared with that in the WT (Figures 2B,C). Taken together, these findings indicate that *NRP1* gene and protein expression levels are significantly increased in the brains of aged AD mice.

### Severe Alzheimer’s Disease Patients Are Highly Expressed With *Nrp1*

**FIGURE 3:**
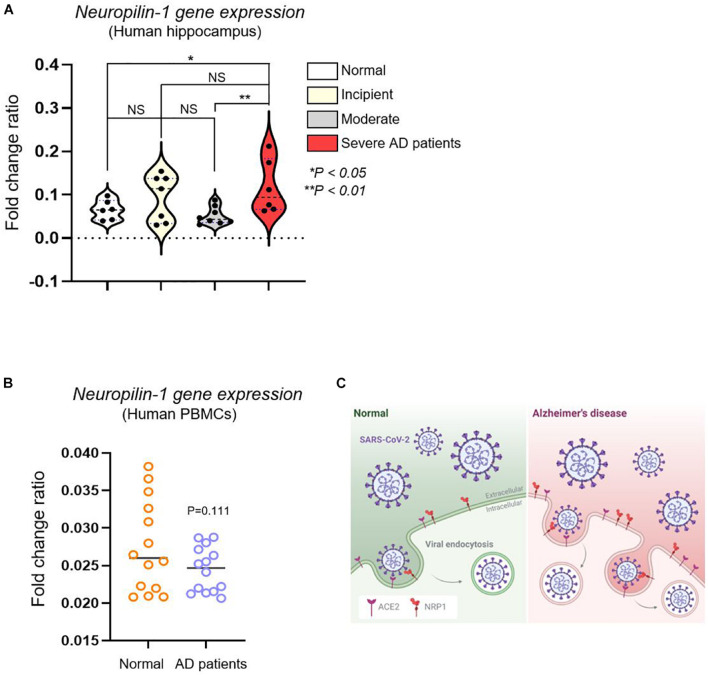
*In silico* analysis of *Nrp1* gene expression in human hippocampus and PBMCs from AD patients. **(A)** *Nrp1* expression is significantly increased in the human hippocampus of severe AD patients compared with that in the control group (179%). No statistical difference is observed when WT is compared to incipient and moderate AD patients. Normal control group *n* = 6, incipient group *n* = 7, moderate group *n* = 8, and severe group *n* = 6. Statistical differences were assessed using *post hoc* test after one-way ANOVA. **(B)** *Nrp1* expression in PBMCs from AD patients is not statistically different from that in the control group. Normal elderly control, female *n* = 7 and male *n* = 7; AD patient group, female *n* = 7 and male *n* = 7. Statistical differences were assessed using unpaired *t*-test. **(C)** Schematic model of NRP1- and ACE2-mediated SARS-CoV-2 infection in AD. NRP1 and ACE2 mediate SARS-CoV-2 binding to the cell membrane and, consequently, infection. Because these two receptors are highly expressed in AD patients, these individuals may be more sensitive to SARS-CoV-2 infection.

Having found increased *Nrp1* gene expression in the brains of AD mice, we next performed *Nrp1* gene expression profiling of brains and PBMCs from human patients with different stages of AD (Supplementary Table 1). To identify the fold change of the ratio for *Nrp1* gene from AD patients, we performed *in silico* analysis using the GSE1297 and GSE4296 microarray data set. Patients with severe AD showed significantly upregulated *Nrp1* gene expression (179%) compared with the control group (individuals without AD), whereas incipient and moderate AD patients did not show increases in brain *Nrp1* gene expression (Figure 3A). Interestingly, we did not find differences in PBMC *Nrp1* gene expression between any of the groups (Figure 3B). These data correlate with results from the AD murine model. Together, the results demonstrate that NRP1 mRNA and protein expression is significantly elevated in the brains of late-stage AD patients.

## Discussion

Since the beginning of the COVID-19 pandemic, there have been significant efforts to identify unique SARS-CoV-2–associated proteins that could serve as targets for novel vaccines or therapeutic agents. Despite notable studies suggesting the possibility of developing other COVID-19-targeted drugs, the first-generation drugs have mostly focused on the viral spike protein receptor ACE2 (Yin et al., 2020). As high-throughput genomic studies begin to define the abnormal expression of individual DNA in particular diseases, it may become possible to rationally determine disease-specific gene expression and, thus, establish biomarkers for risk prediction in older people with complications, such as AD. Recently, we showed the increase of ACE2 expression in an elderly group with AD; therefore, our *in silico* analysis accurately predicts high risk for SARS-CoV-2 infection in elderly patients with AD (Lim et al., 2020). In addition, our research scheme may be useful for predicting the risk of AD in patients with SARS-CoV-2 infection.

Our findings have implications for the prevention and treatment of SARS-CoV-2 infection in elderly patients with AD. First, both *Ace2* and *Nrp1* are preferentially expressed in the brain, and their expression level may determine the sensitivity to SARS-CoV-2 infection (Figure 3C). Interestingly, it was recently suggested that differences in cytokines, such as IL-1β and TNF-α, are less pronounced in peripheral blood in SARS-CoV-2 infection (Totura and Baric, 2012; Tincati et al., 2020). Second, in addition to *Ace2*, *Nrp1* expression was also upregulated in patients with severe AD. Although predictive immune biomarkers are suggested for the clinical treatment of COVID-19 (Fouladseresht et al., 2020), our high-throughput analysis–based approach would probably provide an accurate prediction of SARS-CoV-2 risk in elderly AD patients. Notably, *Ace2* gene expression gradually increased with the severity of AD symptoms (from incipient to severe stage) (Lim et al., 2020), whereas elevated *Nrp1* gene expression was only present in the severe AD patient group (Figures 1B,C). This result indicates that ACE2 may be a more fundamental gene for SARS-CoV-2 infection compared with NRP1.

Recently, the spread of SARS-CoV-2 infection has accelerated worldwide. Efforts on the clinical treatment of SARS-CoV-2 infection are concentrated on the development of vaccines and drugs, including gene therapy (Chugh et al., 2020). To our knowledge, this is the first study examining NRP1 expression in AD patients and reporting its higher expression these individuals. Moreover, it reveals the importance of determining SARS-CoV-2 spike protein receptor gene expression. Our gene profiling could potentially be used to predict the risk for SARS-CoV-2 infection in elderly AD patients.

## Data Availability Statement

The datasets presented in this study can be found in online repositories. The names of the repository/repositories and accession number(s) can be found in the article/Supplementary Material.

## Ethics Statement

All animal experiments performed in this study were reviewed and approved by the IACUC Committee at Korea Brain Research Institute (IACUC-20-00018). Written informed consent was obtained from the owners for the participation of their animals in this study. Written informed consent was obtained from the individual(s) for the publication of any potentially identifiable images or data included in this article.

## Author Contributions

J-YJ and K-HL designed the research. K-HL, SY, S-HK, and J-YJ wrote the manuscript, performed the research, discussed the results, and commented on the manuscript. All authors contributed to the article and approved the submitted version.

## Conflict of Interest

The authors declare that the research was conducted in the absence of any commercial or financial relationships that could be construed as a potential conflict of interest.

## Publisher’s Note

All claims expressed in this article are solely those of the authors and do not necessarily represent those of their affiliated organizations, or those of the publisher, the editors and the reviewers. Any product that may be evaluated in this article, or claim that may be made by its manufacturer, is not guaranteed or endorsed by the publisher.

## References

[B1] Abdullah A. Akhand S. S. Paez J. S. P. Brown W. Pan L. Libring S. (2020). Epigenetic targeting of neuropilin-1 prevents bypass signaling in drug-resistant breast cancer. *Oncogene* 40 322–333. 10.1038/s41388-020-01530-6 33128042 PMC7808937

[B2] Bianchetti A. Rozzini R. Guerini F. Boffelli S. Ranieri P. Minelli G. (2020). Clinical presentation of COVID19 in dementia patients. *J. Nutr. Health Aging* 24 560–562. 10.1007/s12603-020-1389-1 32510106 PMC7227170

[B3] Cantuti-Castelvetri L. Ojha R. Pedro L. D. Djannatian M. Franz J. Kuivanen S. (2020). Neuropilin-1 facilitates SARS-CoV-2 cell entry and infectivity. *Science* 370 856–860. 10.1126/science.abd2985 33082293 PMC7857391

[B4] Carstensen L. L. Shavit Y. Z. Barnes J. T. (2020). Age advantages in emotional experience persist even under threat from the COVID-19 pandemic. *Psychol. Sci.* 31 1374–1385. 10.1177/0956797620967261 33104409 PMC13171095

[B5] Chugh H. Awasthi A. Agarwal Y. Gaur R. K. Dhawan G. Chandra R. (2020). A comprehensive review on potential therapeutics interventions for COVID-19. *Eur. J. Pharmacol.* 890 173741. 10.1016/j.ejphar.2020.173741 33227287 PMC7677683

[B6] Daly J. L. Simonetti B. Klein K. Chen K. E. Williamson M. K. Anton-Plagaro C. (2020). Neuropilin-1 is a host factor for SARS-CoV-2 infection. *Science* 370 861–865. 10.1126/science.abd3072 33082294 PMC7612957

[B7] Davies J. Randeva H. S. Chatha K. Hall M. Spandidos D. A. Karteris E. (2020). Neuropilin-1 as a new potential SARS-CoV-2 infection mediator implicated in the neurologic features and central nervous system involvement of COVID-19. *Mol. Med. Rep.* 22 4221–4226. 10.3892/mmr.2020.11510 33000221 PMC7533503

[B8] Ding Q. Shults N. V. Harris B. T. Suzuki Y. J. (2020). Angiotensin-converting enzyme 2 (ACE2) is upregulated in Alzheimer’s disease brain. *bioRxiv* [Preprint]. 10.1101/2020.10.08.331157 33567524 PMC7914443

[B9] Fouladseresht H. Doroudchi M. Rokhtabnak N. Abdolrahimzadehfard H. Roudgari A. Sabetian G. (2020). Predictive monitoring and therapeutic immune biomarkers in the management of clinical complications of COVID-19. *Cytokine Growth Factor Rev*. 58 32–48. 10.1016/j.cytogfr.2020.10.002 33199179 PMC7544568

[B10] Fu L. Wang B. Yuan T. Chen X. Ao Y. Fitzpatrick T. (2020). Clinical characteristics of coronavirus disease 2019 (COVID-19) in China: a systematic review and meta-analysis. *J. Infect.* 80 656–665. 10.1016/j.jinf.2020.03.041 32283155 PMC7151416

[B11] Hu B. Guo H. Zhou P. Shi Z.-L. (2020). Characteristics of SARS-CoV-2 and COVID-19. *Nat. Rev. Microbiol*. 19 141–154. 10.1038/s41579-020-00459-7 33024307 PMC7537588

[B12] Kumanogoh A. Kikutani H. (2013). Immunological functions of the neuropilins and plexins as receptors for semaphorins. *Nat. Rev. Immunol.* 13 802–814. 10.1038/nri3545 24319778

[B13] Lim K. H. Joo J. Y. (2020). Predictive potential of circulating Ube2h mRNA as an E2 ubiquitin-conjugating enzyme for diagnosis or treatment of Alzheimer’s disease. *Int. J. Mol. Sci.* 21:3398. 10.3390/ijms21093398 32403399 PMC7246987

[B14] Lim K. H. Yang S. Kim S. H. Joo J. Y. (2020). Elevation of ACE2 as a SARS-CoV-2 entry receptor gene expression in Alzheimer’s disease. *J. Infect.* 81 e33–e34. 10.1016/j.jinf.2020.06.072 32619698 PMC7834154

[B15] Liu K. Chen Y. Lin R. Han K. (2020). Clinical features of COVID-19 in elderly patients: a comparison with young and middle-aged patients. *J. Infect.* 80 e14–e18. 10.1016/j.jinf.2020.03.005 PMC7102640 32171866

[B16] Liu P.-P. Xie Y. Meng X.-Y. Kang J.-S. (2019). History and progress of hypotheses and clinical trials for Alzheimer’s disease. *Signal Trans. Targeted Ther.* 4:29. 10.1038/s41392-019-0063-8 31637009 PMC6799833

[B17] Masters C. L. Bateman R. Blennow K. Rowe C. C. Sperling R. A. Cummings J. L. (2015). Alzheimer’s disease. *Nat. Rev. Dis. Primers* 1:15056. 10.1038/nrdp.2015.56 27188934

[B18] Mayi B. S. Leibowitz J. A. Woods A. T. Ammon K. A. Liu A. E. Raja A. (2021). The role of Neuropilin-1 in COVID-19. *PLoS Pathog.* 17:e1009153. 10.1371/journal.ppat.1009153 33395426 PMC7781380

[B19] Rahman M. A. Islam K. Rahman S. Alamin M. (2020). Neurobiochemical cross-talk between COVID-19 and Alzheimer’s disease. *Mol. Neurobiol*. 58 1017–1023. 10.1007/s12035-020-02177-w 33078369 PMC7571527

[B20] Singh D. D. Han I. Choi E.-H. Yadav D. K. (2020). Recent advances in pathophysiology, drug development and future perspectives of SARS-CoV-2. *Front. Cell Dev. Biol.* 8:580202. 10.3389/fcell.2020.580202 33240881 PMC7677140

[B21] Song E. Zhang C. Israelow B. Lu-Culligan A. Prado A. V. Skriabine S. (2020). Neuroinvasion of SARS-CoV-2 in human and mouse brain. *bioRxiv* [Preprint]. 10.1084/jem.20202135 33433624 PMC7808299

[B22] Teesalu T. Sugahara K. N. Kotamraju V. R. Ruoslahti E. (2009). C-end rule peptides mediate neuropilin-1-dependent cell, vascular, and tissue penetration. *Proc. Natl. Acad. Sci. U.S.A.* 106 16157–16162. 10.1073/pnas.0908201106 19805273 PMC2752543

[B23] Tincati C. Cannizzo E. S. Giacomelli M. Badolato R. D’arminio Monforte A. Marchetti G. (2020). Heightened circulating interferon-inducible chemokines, and activated pro-cytolytic Th1-cell phenotype features Covid-19 aggravation in the second week of illness. *Front. Immunol.* 11:580987. 10.3389/fimmu.2020.580987 33193384 PMC7606391

[B24] Totura A. L. Baric R. S. (2012). SARS coronavirus pathogenesis: host innate immune responses and viral antagonism of interferon. *Curr. Opin. Virol.* 2 264–275. 10.1016/j.coviro.2012.04.004 22572391 PMC7102726

[B25] Verkhratsky A. Li Q. Melino S. Melino G. Shi Y. (2020). Can COVID-19 pandemic boost the epidemic of neurodegenerative diseases? *Biol. Direct* 15:28. 10.1186/s13062-020-00282-3 33246479 PMC7691955

[B26] Wu J. T. Leung K. Bushman M. Kishore N. Niehus R. De Salazar P. M. (2020). Estimating clinical severity of COVID-19 from the transmission dynamics in Wuhan. China. *Nat. Med.* 26 506–510. 10.1038/s41591-020-0822-7 32284616 PMC7094929

[B27] Yin S. Tong X. Huang A. Shen H. Li Y. Liu Y. (2020). Longitudinal anti-SARS-CoV-2 antibody profile and neutralization activity of a COVID-19 patient. *J. Infect.* 81 e31–e32. 10.1016/j.jinf.2020.06.076 32622905 PMC7330586

