# Supplementary material for: Identifying New COVID-19 Receptor Neuropilin-1 in Severe Alzheimer’s Disease Patients Group Brain Using Genome-Wide Association Study Approach
Source: Front Genet. 2021 Oct 21;12:741175. doi: 10.3389/fgene.2021.741175 (PMC8566993; doi:10.3389/fgene.2021.741175)
Supplement: Supplementary file 1 [file Table_1.DOCX]

**Supplementary table 1. Description for AD patients from GSE1297 microarray dataset.**

| **Group** | **Age** | **Sex** | **PMI** | **MMSE** | **BRAAK** |
| --- | --- | --- | --- | --- | --- |
| Control | 85 | Male | 2 | 30 | 3 |
|  | 92 | Female | 2.45 | 26 | 2 |
|  | 80 | Male | 3 | 26 | 4 |
|  | 75 | Male | 3.35 | 29 | 1 |
|  | 97 | Female | 2.75 | 29 | 3 |
|  | 95 | Male | 1.75 | 28 | 1 |
| Incipient | 95 | Female | 6.17 | 29 | 6 |
|  | 101 | Female | 1.35 | 25 | 5 |
|  | 83 | Female | 2.25 | 26 | 5 |
|  | 88 | Male | 2.75 | 24 | 6 |
|  | 91 | Female | 4 | 25 | 3 |
|  | 88 | Male | 2.75 | 20 | 5 |
|  | 97 | Female | 4 | 21 | 5 |
| Moderate | 86 | Female | 4.25 | 15 | 6 |
|  | 85 | Female | 2.92 | 18 | 6 |
|  | 89 | Female | 2.63 | 15 | 5 |
|  | 83 | Female | 2.53 | 17 | 5 |
|  | 82 | Female | 2.75 | 18 | 5 |
|  | 79 | Female | 3 | 18 | 6 |
|  | 81 | Male | 3.5 | 17 | 6 |
|  | 82 | Male | 4 | 14 | 6 |
| Severe | 85 | Male | 2.5 | 4 | 6 |
|  | 65 | Female | 3.05 | 5 | 6 |
|  | 93 | Male | 3.08 | 11 | 6 |
|  | 79 | Female | 3 | 11 | 6 |
|  | 94 | Female | 2.72 | 2 | 5 |
|  | 79 | Female | 3.67 | 2 | 6 |

*PMI: Postmortem interval; MMSE: Mini mental status examination; BRAAK: Braak stage
